# Metacommunity Theory and Metabarcoding Reveal the Environmental, Spatial and Biotic Drivers of Meiofaunal Communities in Sandy Beaches

**DOI:** 10.1111/mec.17733

**Published:** 2025-03-20

**Authors:** Maximilian Pichler, Simon Creer, Alejandro Martínez, Diego Fontaneto, Willem Renema, Jan‐Niklas Macher

**Affiliations:** ^1^ Theoretical Ecology University of Regensburg Regensburg Germany; ^2^ Molecular Ecology and Evolution Group, School of Natural and Environmental Sciences Bangor University Bangor Gwynedd UK; ^3^ National Research Council of Italy (CNR) Water Research Institute (IRSA) Verbania Pallanza Italy; ^4^ National Biodiversity Future Center (NBFC) Palermo Italy; ^5^ Naturalis Biodiversity Center, Marine Biodiversity Leiden the Netherlands; ^6^ Institute for Biodiversity and Ecosystem Dynamics University of Amsterdam Amsterdam the Netherlands; ^7^ Department of Environmental Biology Institute of Environmental Sciences (CML), Leiden University Leiden the Netherlands

**Keywords:** community ecology, metabarcoding, metacommunities, niche modelling, species interactions

## Abstract

Understanding the processes that shape community assembly is a critical focus of ecology. Marine benthic meiofauna, microscopic invertebrates inhabiting sediment environments, play important roles in ecosystem functioning but have been largely overlooked in metacommunity studies due to the lack of community data. In this study, we quantify the relative contributions of environmental filtering, spatial processes, and biotic associations in structuring meiofaunal communities. We applied Generalised Dissimilarity Modelling (GDM) and Joint Species Distribution Modelling (JSDM) to an extensive metabarcoding dataset comprising 550 samples collected from sandy beaches along over 650 km of the Dutch and German North Sea coast. Our findings reveal that biotic associations, followed by environmental factors, particularly the distance from the low tide line and sediment grain size, are primary drivers of meiofauna community turnover, highlighting the influence of sharp environmental gradients. Spatial factors indicating dispersal limitations have no major impact on community composition, supporting the assumption that microscopic organisms have strong dispersal capabilities. JSDM results demonstrate that while species sorting is a key driver of community assembly, environmental factors are most important in environmentally distinct (‘extreme’) sites, whereas biotic associations significantly shape community assembly in both environmentally similar and dissimilar habitats, emphasising the need to incorporate species interactions into models of community assembly. By providing insights into the drivers of meiofaunal community structure, our study highlights the importance of environmental gradients and biotic associations in shaping biodiversity patterns and underscores the potential for similar approaches to enhance understanding of other ecosystems with small, highly diverse, but understudied taxa.

## 1 Introduction

Understanding the processes that shape community assembly is a central theme in ecology, with significant implications for biodiversity conservation and ecosystem functioning (Holyoak et al. 2005; Leibold et al. 2004; Vellend 2020). Metacommunity theory provides a framework for exploring how species sorting, dispersal, biotic interactions and ecological drift interact to influence community composition, and how these mechanisms connect local communities across spatial scales (Leibold and Chase 2018). Despite extensive research in various ecosystems, debate continues about the relative contributions of these processes and the conditions under which each mechanism prevails (Brown et al. 2017; Cottenie 2005; Logue et al. 2011).

Metacommunity theory has been widely applied across diverse taxa (Heino et al. 2017; Miller et al. 2018; Nemergut et al. 2013; Terry et al. 2022). However, a knowledge gap remains in the relative importances of metacommunity processes, particularly in highly dynamic, physically controlled environments, where spatial continuity at larger scales coexists with sharp local environmental gradients and frequent disturbances (Datry, Bonada, et al. 2016; Datry, Melo, et al. 2016; Defeo and McLachlan 2025). This interplay between large‐scale connectivity and fine‐scale heterogeneity may reveal distinct locally changing community assembly patterns, challenging traditional metacommunity models that have shown limited capabilities to infer assembly processes (Ovaskainen et al. 2019; Guzman et al. 2022; Leibold et al. 2022; Cai et al. 2024).

Marine meiofauna provide an ideal system for testing how metacommunity dynamics operate in spatially continuous but environmentally heterogeneous conditions. Meiofaunal communities inhabit interstitial spaces within sediments, experiencing steep environmental gradients over small spatial scales. Meiofaunal community structure is shaped by environmental factors, including sediment composition (Fegley et al. 2020; Hulings and Gray 1976; Maria et al. 2018; McLachlan and Defeo 2017) hydrodynamics (Kotwicki et al. 2014; McLachlan et al. 2018; Rodríguez et al. 2003), microhabitat patchiness (Leasi et al. 2016), and intertidal gradients (Ape et al. 2017; Hua et al. 2016; Pereira et al. 2017). Spatial processes, including dispersal limitation (Leibold and Chase 2018) due to the absence of planktonic stages and passive transport via hydrodynamic forces, also influence community structure (Cerca et al. 2018; Derycke et al. 2008; Worsaae et al. 2019). However, the relative contributions of species sorting (niche), dispersal limitation, and neutral processes (neutral theory, Hubbell 2011) remain poorly understood, and clarifying their roles is critical to predicting meiofaunal responses to environmental change (Giere and Schratzberger 2023; Leasi et al. 2021; Zeppilli et al. 2015) but also for finally shedding light on their relative contribution, depending on environmental and spatial distinctiveness (Cai et al. 2024), to compositional variability.

Meiofauna are crucial to benthic ecosystem functioning, with their responses potentially affecting higher trophic levels and overall ecosystem health. These invertebrates range in size from ~20–40 μm to 1 mm, depending on the definition (Giere 2009; Ptatscheck et al. 2020). They play critical roles in nutrient cycling and act as a vital link between primary producers and higher trophic levels (Maria et al. 2012; Menn 2002; Schratzberger and Ingels 2018; van Der Heijden et al. 2020), yet our understanding of the drivers of their compositional variation is lacking. Sandy beaches provide a natural setting where meiofaunal communities experience strong environmental gradients and frequent disturbances, making them an ideal system to study how metacommunity processes operate in highly dynamic environments, and metacommunity theory can improve our understanding of these vulnerable ecosystems (Defeo et al. 2009; Schlacher et al. 2007) with direct relevance for conservation and coastal ecosystem management.

Recent advancements in molecular techniques, particularly metabarcoding, enable cost‐effective, large‐scale assessments of meiofaunal diversity (Bellisario et al. 2021; Brannock et al. 2018, 2023; de Faria et al. 2018; Gielings et al. 2021). Coupled with ecological modelling, these approaches offer new opportunities to disentangle how species sorting, dispersal limitation, and biotic interactions together shape meiofaunal communities in dynamic environments by disentangling the relative importance of environmental, spatial, and biotic factors (Leibold et al. 2022). However, integrating biotic interactions into models is challenging, especially for highly diverse taxa. Generalised Dissimilarity Modelling (GDM) provides insights into community turnover across environmental gradients (Ferrier et al. 2007), and thus the external structure of metacommunities (Leibold et al. 2022). Joint Species Distribution Modelling (JSDM) simultaneously assesses environmental effects, spatial processes, and species co‐occurrence patterns, revealing the internal structure of metacommunities (Ovaskainen and Abrego 2020; Seaton et al. 2024; Tikhonov et al. 2017, 2020). Because co‐occurrence patterns in JSDM can correspond to latent biotic interactions as well as missing environmental variables (Hartig et al. 2024), we refer to them collectively as biotic associations. Recent developments make it feasible to apply JSDMs to large metabarcoding datasets (Cai et al. 2024; Hartig et al. 2024), offering new opportunities for understanding how metacommunities are shaped in highly dynamic habitats.

In this study, we apply GDM and JSDM to an extensive metabarcoding dataset of marine meiofaunal communities collected from sandy beaches along over 650 km of the Dutch and German North Sea coast. By integrating these modelling approaches, we aim to quantify the extent to which biotic associations contribute to community assembly relative to environmental and spatial factors, providing insights into how metacommunity models can help understand communities in continuous yet heterogeneous environments. We hypothesise that species sorting driven by environmental factors (e.g., sediment grain size, beach morphodynamics) is stronger in more extreme environmental sites, in line with metacommunity theory predictions that strong environmental gradients favour species sorting (Leibold et al. 2004; Leibold and Chase 2018). Given the well‐documented influence of sediment characteristics on meiofaunal composition, we expect that species sorting due to environmental factors will dominate in these dynamic habitats. However, dispersal limitation and biotic interactions also influence community assembly, and therefore, we propose an alternative hypothesis that these processes also contribute significantly to meiofaunal community structure, in particular in spatially isolated sites. By integrating biotic associations into metacommunity models, our study refines predictions of community assembly in spatially continuous but environmentally heterogeneous systems, providing insights into biodiversity dynamics in highly dynamic ecosystems.

## 2 Material and Methods

### 2.1 Sampling and Environmental Variable Measurement

We collected meiofauna from 24 sea‐facing, unsheltered sandy beaches of the southern North Sea, covering 650 km of coastline between Zeeland (southern Netherlands) and Sylt (northern Germany). Sampling took place during the summers of 2021 and 2022 (See Table S1 for coordinates and Figure S1A for a map of sampling sites).

**FIGURE 1:**
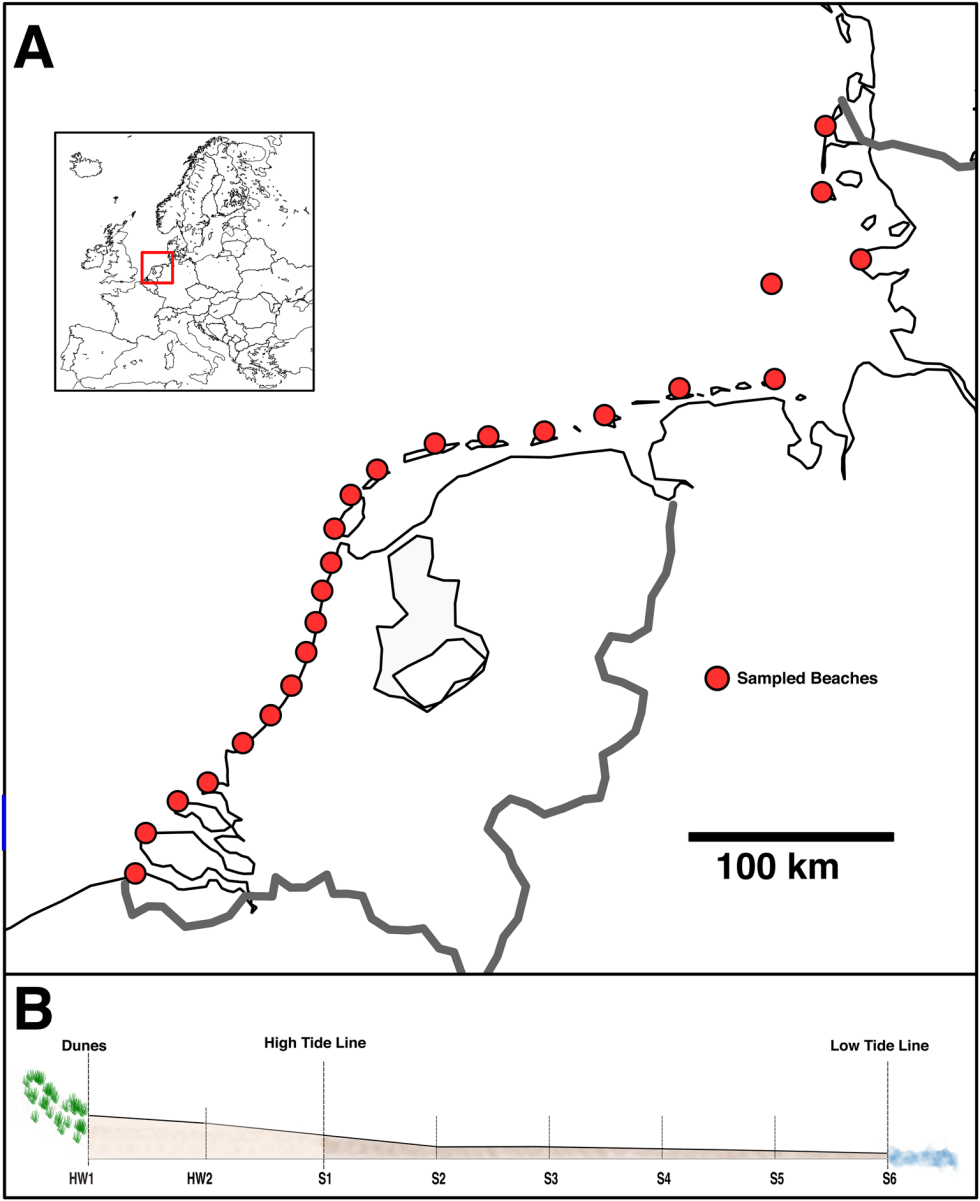
(A) Map of the study area showing the 24 sampled beaches. The small map indicates the location of the study area in Europe. (B) Schematic view of a sampling transect across a beach.

Samples were taken during daytime and at maximum low tide. We sampled along three parallel transects per beach, each with eight sampling sites. The first sample was taken at the foot of the dunes, the second sample halfway between the dunes and the high‐tide line, and six samples were equidistantly spaced from the high‐tide line to the low‐tide line (see Figure 1B). Sampling followed established protocols for the intertidal zone of sandy beaches, including the measurement of beach width from high‐tide line to low‐tide line, measuring the beach slope (in degrees), wave period (in seconds) and breaker height (in metres) (McLachlan et al. 2018). The tidal range was extracted from online databases (www.tide‐forecast.com), and we calculated the Relative Tide Range (RTR) index for each beach (McLachlan et al. 2018). Furthermore, we assessed the beach state (reflective, intermediate, dissipative) At each sampling site, we collected two sediment cores using sterile plastic syringes: one core of 5 cm diameter and a length of 10 cm (volume ≈200 mL), and a second core of 1 cm diameter and a length of 10 cm (volume ≈8 mL). The small sediment core was immediately transferred to a 50 mL Falcon tube, and the large sediment core was transferred to a sterile 1 L plastic bottle. We extracted meiofauna from the large sediment core (≈200 mL) directly on the beach using the MgCl_2_ decantation method (Atherton and Jondelius 2020). We added 500 mL of isosmotic MgCl_2_ solution to the sediment, which anaesthetizes meiofauna and allows their separation from the sediment by decantation. After 5 min, the sediment in the MgCl_2_ solution was carefully swirled 10 times, and the supernatant containing meiofauna was decanted through a 1 mm and 41 μm sieve cascade, as commonly done in beach meiofauna studies (Castro et al. 2021; Haenel et al. 2017; Martínez et al. 2020). The meiofauna fraction retained on the 41 μm sieve was rinsed into sterile 15 mL Falcon tubes and preserved with 10 mL 96% EtOH. All sampling equipment was thoroughly rinsed with ethanol and sterile water after taking each sample to prevent contamination. All samples were transported back to the Naturalis Biodiversity Centre laboratory and stored at −20°C until further processing. Sediment from the smaller core was dried, and the grain size was measured on a LS13320 Particle Size Analyser (Beckman‐Coulter, USA) for the eight samples of the central transect per beach.

### 2.2 DNA Extraction, Amplification and Sequencing

We extracted DNA from dried meiofauna samples after evaporating the ethanol at 50°C overnight in a sterile warming cabinet and transferring the dried samples to 2 mL Eppendorf tubes. DNA extraction was performed using the Macherey Nagel NucleoSpin Soil kit (Macherey Nagel, Düren, Germany) following the standard protocol including bead beating, but with an additional overnight Proteinase K digestion step (50 μL 250 μg/mL ProtK, Thermo Fisher Scientific, Waltham, USA) added to the lysis buffer provided with the kit to improve cell lysis, as done in previous studies on meiofauna (Martínez et al. 2020; Weigand and Macher 2018).

For community metabarcoding, we amplified meiofauna DNA using a two‐step PCR protocol with the widely used LerayXT primers targeting a 313 base pair region of the mitochondrial cytochrome c oxidase I (COI) gene of a broad range of Eukaryota (Collins et al. 2019; Wangensteen et al. 2018). We chose the COI gene due to its strong species‐level resolution, which is important for subsequent joint species distribution modelling (JSDM). Additionally, the availability of an expert‐curated reference database for the study region (Macher et al. 2024) and financial considerations further led to this choice.

The first PCR reaction contained 11.7 μL mQ water, 2 μL Qiagen CL buffer (10×; Qiagen, Hilden, Germany), 0.4 μL MgCl2 (25 mM; Qiagen), 0.8 μL Bovine Serum Albumin (BSA, 10 mg/mL), 0.4 μL dNTPs (2.5 mM), 0.2 μL Qiagen Taq (5 U/μL), 1 μL of each nextera‐tailed primer (10 pMol/μL), and 2.5 μL of DNA template. PCR amplification involved an initial denaturation at 96°C for 3 min, followed by 30 cycles of denaturation for 15 s at 96°C, annealing at 50°C for 30 s, and extension for 40 s at 72°C, concluding with a final extension at 72°C for 5 min. We processed six negative controls (containing Milli‐Q water instead of DNA template; Milli‐Q, Merck, Kenilworth, USA) alongside the samples to check for potential contamination.

After the first PCR, samples were cleaned with AMPure beads (Beckman Coulter, Brea, United States) at a 0.9:1 ratio according to the protocol to remove short fragments and primer dimers. The second PCR involved amplification with individually tagged primers, following the same protocol as above and using the PCR product from the first PCR as the template, but reducing the PCR cycle number to 10. We measured DNA concentrations using the Fragment Analyzer (Agilent Technologies, Santa Clara, CA, USA) with the High Sensitivity Kit and pooled samples equimolarly. The final library was cleaned with AMPure beads as described above and sent for sequencing on three Illumina MiSeq runs (2 × 300 bp read length) at Baseclear (Leiden, The Netherlands).

### 2.3 Bioinformatic Processing of Community Metabarcoding Data

We processed the raw metabarcoding reads using APSCALE (Buchner et al. 2022) with the following settings: maximum differences in percentage: 20; minimum overlap: 50, minimum sequence length: 310 bp; maximum read length: 316 bp, minimum size to pool: 20 sequences. Sequences were clustered into Operational Taxonomic Units (OTUs) with a sequence similarity threshold of 97%. We chose to analyse OTUs instead of Amplicon Sequence Variants (ASVs) to obtain units more comparable to species‐level resolution, which was necessary for the subsequent JSDM analyses. To account for potential low‐level contamination or tag jumping common on Illumina platforms (Schnell et al. 2015), we removed OTUs with an abundance of < 0.03% of reads per sample, following a common subsetting approach in metabarcoding studies (Brasseur et al. 2023; Hupało et al. 2021). We removed 11 samples with < 3000 reads per sample during bioinformatic processing, retaining samples for which 1 read corresponds to > 0.03% of the total reads number. We performed taxonomic assignment using NCBI GenBank expanded with meiofauna COI barcodes generated as part of associated taxonomic workshops in Leiden (Macher et al. 2024). Taxonomic ranks were assigned to OTUs using established identity thresholds: > 97%: species, > 95%: genus, > 90%: family, > 85%: order (Macher et al. 2023). Taxonomic annotation of the reads present in the six negative controls (5211 reads total) showed that only two OTUs (OTU222, *Navicula*, a diatom, with 2728 reads; and OTU219, *Homo sapiens* , with 2653 reads) were dominant in negative controls. No meiofauna sequences were found in the negative controls, and therefore we did not remove any OTUs from the dataset to be analysed.

Subsequently, OTUs that were assigned with < 85% identity to a reference or identified as non‐meiofauna taxa were excluded from further analyses. We merged the three sampling units per tidal level per beach into one composite sample to account for potential variability within tidal levels, resulting in 190 composite samples. Following this, we retained only OTUs that were present in at least 10 samples, following the recommendation of Cai et al. (2024), since sjSDM can be sensitive to false negative occurrences.

### 2.4 Environmental Variable Selection and Generalised Dissimilarity Modelling

We measured the following environmental variables: Grain Size (average per sample, in μm), Distance from Low Tide Level (in percentage, with low tide = 0 and the sample closest to the dunes = 100%), Beach Width (in metres), Salinity of the Surface Water (in ppm), Spring Tide Range (in metres), Average Beach Slope (in degrees). Further, we obtained the following variables from the Bio‐ORACLE v2 database containing marine data for modelling (Assis et al. 2018): Mean Surface Water Temperature (°C), Mean Surface Phosphate (mmol m^−3^), Mean Surface Nitrate (mmol m^−3^), Surface Dissolved Oxygen (mmol m^−3^), Mean Surface Salinity (PSS). We also downloaded bioclimatic variables from the WorldClim 2 database (Fick and Hijmans 2017), which contains data for terrestrial environments: Annual Mean Temperature (°C) and Annual Precipitation (mm). All variables were extracted from the data layers using QGIS v. 3.36 (http://qgis.org/). We calculated the pairwise correlation of the variables using the cor function in R and removed variables that showed a correlation coefficient > 0.7. Following this, the following variables were retained: Grain size, Distance from Low Tide, Beach width, Spring Tide Range, Average Beach Slope, Mean Primary Production, Mean Surface Salinity, Average Annual Temperature. We then used the collinearity diagnostic variance inflation factor, implemented in the ‘vifstep’ function of the R package usdm (Naimi 2012) with a threshold of 0.5, which excluded the variables Mean Primary Production and Beach Width. The retained variables, which all showed a VIF score < 2, were Grain Size, Distance from Low Tide, Spring Tide Range, Average Beach Slope, Mean Surface Salinity and Average Annual Temperature.

We used the R package ‘gdm’ (Manion et al. 2017) to calculate the GDM based on a Jaccard distance matrix of community similarity. The environmental variables were provided as predictors, and geography, based on a distance matrix, was included in the calculations. The GDM plots were generated with the plot function from the gdm package.

### 2.5 Scalable Joint Species Distribution Models (sjSDM)

We fitted the sJSDM model using the ‘sjsdm’ package v.1.0.5 (Pichler and Hartig 2021) in R. We modelled the spatial relationships between sites as a polynomial of second order of the scaled coordinates (trend surface model) (Dormann et al. 2007; Griffith and Peres‐Neto 2006). Given that read abundance is not a reliable estimate of organism abundance, all OTUs were converted to presence/absence and fitted with a binomial model with probit link. We modelled the responses to environmental gradients as linear responses, set the learning rate scheduler to 10, the reduce factor to 0.9 (optimiser parameters that help with the convergence), and ran the model with 500 iterations. We visualised the estimated effects of the six environmental covariables on OTU prevalence using the model plot function in the sjsdm package, with OTUs coloured and sorted by meiofaunal groups. Further, we plotted the biotic associations of OTUs (the estimated variance–covariance matrix, normalised to a correlation matrix), sorted by meiofaunal groups, by extracting the correlation coefficients per pair of OTUs from the sjSDM model output and calculating pairwise correlation plots after filtering to the 2.5% most negative and positive values. We used variation partitioning to calculate the importance of the three assembly processes ‘environment’, ‘space’ and ‘biotic association’ per sampling site and per OTU, the internal structure (Leibold et al. 2022), and we regressed the individual sample *R* ^2^ values for ‘environment’, ‘space’ and ‘biotic covariance’ against the environmental distinctiveness of samples using quantile regression (50% quantile) because of the non‐normal distribution of the *R* ^2^ values and potential outliers. We also tested the same processes against spatial distinctiveness of samples and also against OTU richness per sample. Furthermore, we tested each environmental covariate individually.

## 3 Results

### 3.1 Bioinformatics and OTU Annotation

Following bioinformatic processing with APSCALE and quality filtering, we retained 14,822,456 mitochondrial cytochrome c oxidase I (COI) sequences and 566 Operative Taxonomic Units (OTUs). Following taxonomic annotation using NCBI GenBank and meiofauna reference barcodes reported in (Macher et al. 2024), we retained 11,029,442 sequences of 127 OTUs. Of these, 42 were Nematoda, 21 Copepoda, 16 Clitellata, 12 Polychaeta, 9 Gastrotricha, 8 Platyhelminthes, 7 Acoela, 4 Collembola, 3 Rotifera, 2 Branchiopoda, 1 Nemertea, 1 Tardigrada and 1 Arachnida.

### 3.2 Generalised Dissimilarity Models

**FIGURE 2:**
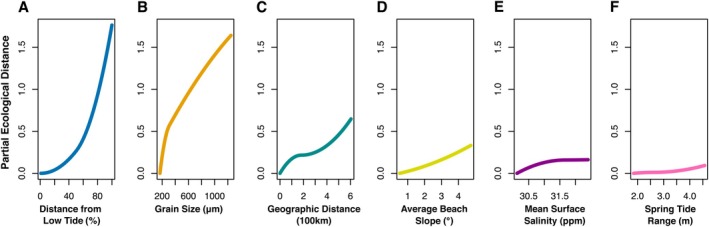
I‐splines (partial ecological distance) from generalised dissimilarity modelling (GDM). The slope of the partial ecological distance indicates the rate of compositional turnover and how it changes with increasing variable values. (A) Distance from Low Tide, (B) Sediment Grain Size, (C) Geographic Distance, (D) Average Beach Slope, (E) Mean Surface Salinity, (F) Spring Tide Range.

The GDM explained 43.2% of the deviance, indicating the proportion of variation in community turnover accounted for by the model. The null deviance was 2223.6, and the GDM deviance was 1262.2. The intercept value was 0.9. The most influential predictor was Distance from low tide, with a sum of I‐spline coefficients of 1.8, with the strongest change occurring at distances corresponding to the high tide line (Figure 2A). This was followed by Grain Size (sum of coefficients: 1.6), with the strongest change occurring between 100 and 400 μm (Figure 2B). The third most influential factor was Geographic Distance (sum of coefficients: 0.7), with a strong change in community turnover at distances between 0 and 100 km, a less pronounced change between distances of 100–400 km, followed by a stronger change up to 600 km (Figure 2C). This was followed by Average Beach Slope (sum of coefficients: 0.3), with community turnover linearly increasing from flat to steep slopes (Figure 2D), and Mean Surface Salinity (sum of coefficients: 0.2), which influenced community turnover mostly between 30 and 31 ppm (Figure 2E). Spring Tide Range (sum of coefficients: 0.1) influenced community turnover mostly at higher ranges above 3.5 m (Figure 2F). Mean Annual Temperature showed a sum of coefficients of 0.0 and did not significantly influence community turnover.

### 3.3 Scalable Joint Species Distribution Models

The sJSDM model resulted in a log likelihood of −5470.78 and an *R* ^2^ value of 0.41.

### 3.4 Estimated Ecological Niches

**FIGURE 3:**
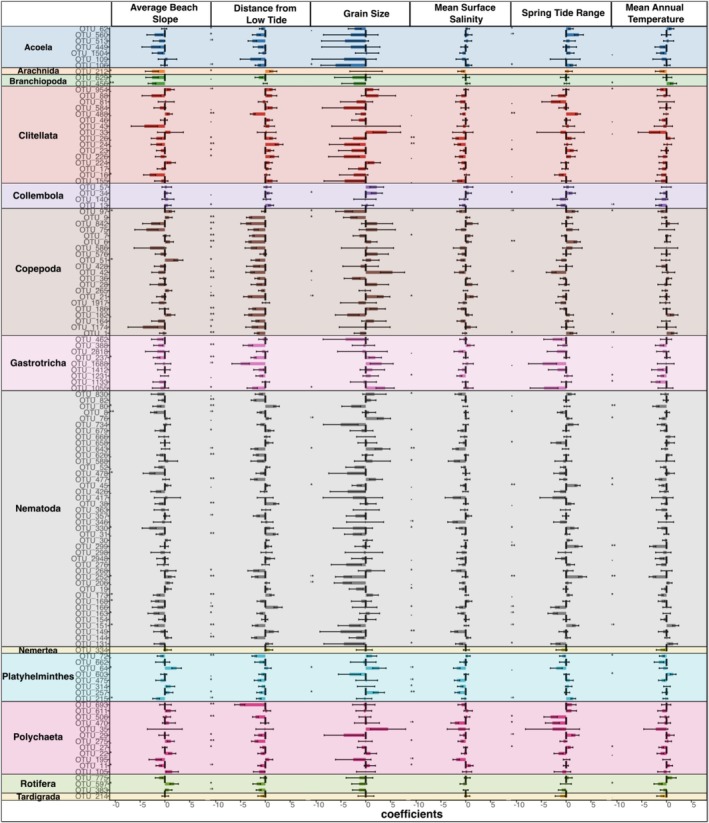
Estimated ecological niches of meiofauna OTUs. Horizontal bars show the magnitudes, directions, and standard errors of the coefficients of each of the six environmental covariates for each meiofaunal OTU, sorted by meiofaunal group. All covariates were normalised before fitting. Colours indicate meiofaunal groups. Asterisks indicate the significance of the effect.

The distance of the sampling site from the low tide level was the most influential factor on OTU prevalence and inferred realised ecological niches. OTUs from Acoela, Branchiopoda, Copepoda, Gastrotricha, Platyhelminthes, Polychaeta, Rotifera and Tardigrada were more prevalent closer to the low tide level, indicating a realised ecological niche in more marine conditions. In contrast, OTUs from Arachnida, Clitellata and Collembola showed higher prevalence further away from low tide, indicating a realised ecological niche in more terrestrial conditions. Nematoda OTUs had mixed responses, with some showing higher prevalence in marine and others in more terrestrial conditions. Most OTUs exhibited a negative relationship with increasing Beach Slope, although this effect was not significant in most cases. Similarly, more OTUs showed a negative relationship with increasing Salinity. Grain size, Spring Tide Range, and Mean Annual Temperature had mixed effects on OTU prevalence, generally lacking significance (Figure 3).

### 3.5 Biotic Associations

Analyses of biotic associations focusing on the top 2.5% of positive and negative correlations revealed many associations between meiofaunal OTUs. For brevity, only the most obvious results are described here. For details, please see Figure S1. Clitellata OTUs showed mostly positive correlations with other Clitellata, negative correlations with Acoela OTUs, and mixed correlations with Nematoda OTUs. Copepoda OTUs were mostly positively correlated with other Copepoda, Polychaeta, and Platyhelminthes OTUs, and showed mixed correlations with Nematoda OTUs. Nematoda OTUs showed both positive and negative correlations within their group and most other meiofauna groups but mostly negative correlations with Gastrotricha OTUs (Figure S1).

### 3.6 The Role of Environmental Distinctiveness for Community Assembly

**FIGURE 4:**
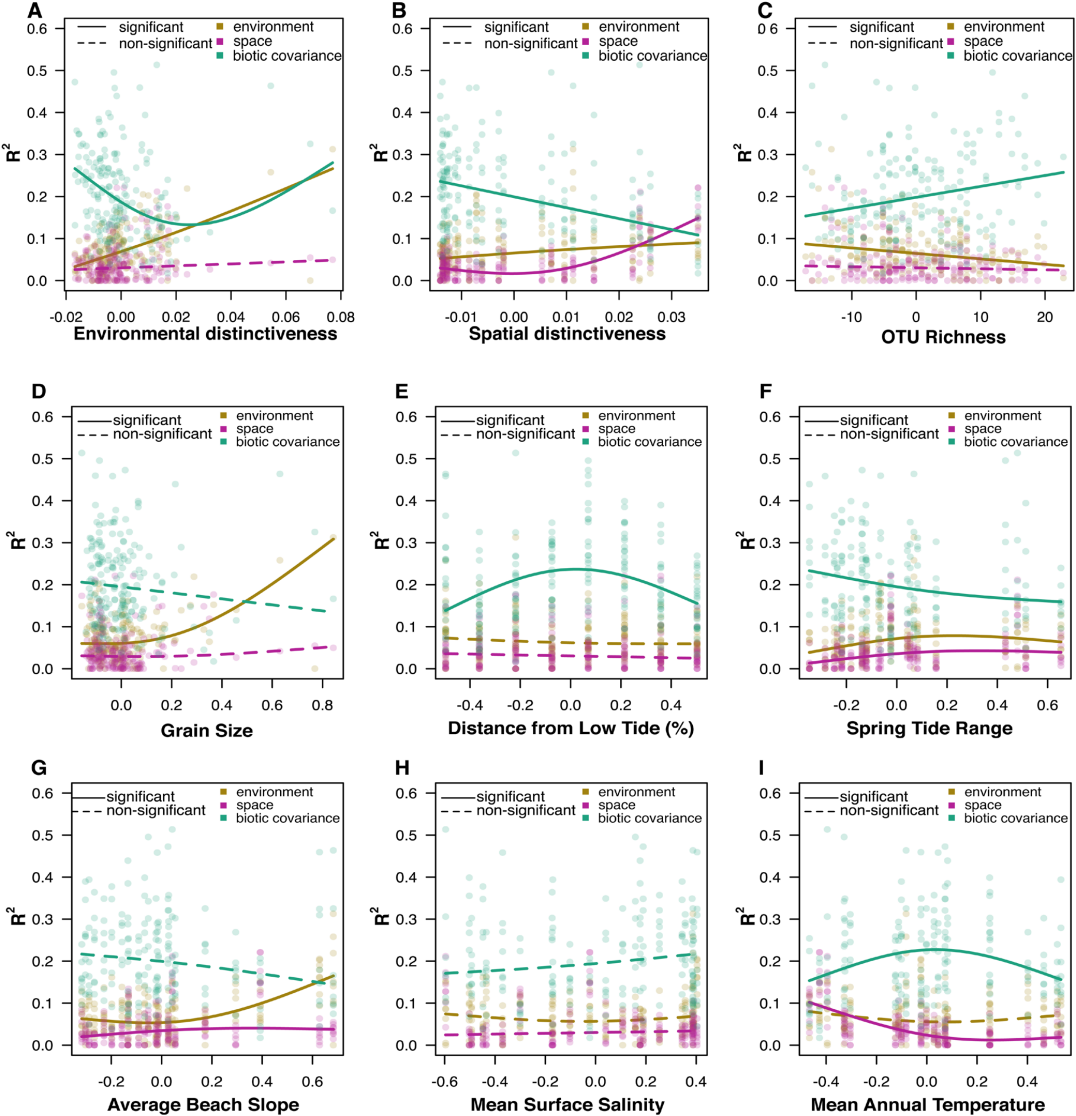
Correlation of the importance of assembly processes with environmental and spatial predictors. Quantile regressions show the importance of the three assembly mechanisms—environment (yellow), space (purple) and biotic covariance (green)—measured by the share of absolute partial *R* ^2^ values per sampling site. (A) Scaled environmental distinctiveness, (B) scaled spatial distinctiveness, (C) scaled OTU richness, (D) grain size, (E) distance from low tide, (F) spring tide range, (G) average beach slope, (H) mean surface salinity and (I) mean annual temperature. Significant effects (*p* < 0.05) are shown as continuous lines, non‐significant effects as dashed lines. All environmental covariates were scaled.

The JSDM analyses of drivers of community assembly showed that as the environmental distinctiveness of sampling sites increased, the *R* ^2^ explained by the environmental component rose significantly and linearly from low to high environmental distinctiveness, indicating stronger species sorting due to environmental differences in sampling sites. The biotic covariance component showed a strong non‐linear response with increasing environmental distinctiveness, dropping from low to medium levels before increasing towards high levels of environmental distinctiveness. This indicates that biotic interactions or similar environmental preferences of meiofaunal OTUs are more important both at low and at high levels of environmental distinctiveness of sampling sites (Figure 4A).

#### 3.6.1 The Role of Spatial Distinctiveness for Community Assembly

With increasing spatial distinctiveness of sampling sites, the *R* ^2^ explained by the spatial component increased significantly from medium to high levels, indicating stronger dispersal barriers between sampling sites that are further apart. At the same time, the environmental component also increased slightly with increasing spatial distinctiveness. The biotic covariance component decreased significantly from low to high levels of spatial distinctiveness (Figure 4B).

#### 3.6.2 The Role of OTU Richness for Community Assembly

With increasing OTU richness per sampling site, the *R* ^2^ explained by biotic covariance showed an increase from low to high OTU richness, indicating stronger biotic associations of OTUs. At the same time, the environmental component decreased significantly with increasing OTU richness (Figure 4C).

#### 3.6.3 Influence of Individual Environmental Covariates on Community Assembly

The *R* ^2^ for the environmental component increased significantly (*p* < 0.05) with increasing Grain Size, indicating that coarser sediments play an important role in meiofauna community assembly (Figure 4D). With increasing Distance from Low Tide, only the biotic association component showed a strong nonlinear relationship, peaking at intermediate distances from low tide (Figure 4E). With increasing Spring Tide Range, we found a significant but minor increase in the *R* ^2^ value of the environmental component, particularly between low and medium values. The spatial component also increased but explained less than the environmental component. The biotic association component declined significantly from low to high Spring Tide Range values (Figure 4F). With increasing Beach Slope, the environmental component increased significantly from intermediate to high values. The spatial component showed a slight increase (Figure 4G). Mean Surface Salinity did not significantly change the environmental, spatial or biotic covariance component (Figure 4H). For the Mean Annual Temperature, we found a decrease in the spatial component from low to intermediate levels of Annual Temperature. The biotic association component showed a non‐linear relationship with increasing temperature, with lowest values at low and high temperatures and a peak at intermediate levels (Figure 4I).

## 4 Discussion

We studied the processes shaping beach meiofauna metacommunities and hypothesised that environmental factors—particularly sediment grain size and beach morphodynamics—are the primary drivers of species sorting and thus the dominant forces structuring meiofaunal communities. Our results largely support our primary hypothesis. The strongest environmental effects were linked to sediment characteristics and tidal gradients. By applying advanced modelling approaches—Generalised Dissimilarity Modelling (GDM) and Joint Species Distribution Modelling (JSDM)‐ we aimed to disentangle the relative contributions of environmental filtering, spatial processes, and biotic interactions in structuring communities of small‐bodied, understudied organisms. Our results confirm that variation in meiofauna communities are the result of locally varying drivers of community assembly and that there is no overall dominant driver.

Our study advances metacommunity theory by addressing the question of how important the different assembly processes (environmental, spatial and biotic factors) are for the individual sites and species, and if we can conclude general assembly rules from these relative effects. Meiofaunal communities inhabiting beach ecosystems are an excellent system for studying these questions because of their exposure to sharp local environmental gradients and frequent disturbances. Our results demonstrate that even in such continuous landscapes, environmental filtering remains important, but biotic associations also play significant roles in shaping communities.

This highlights how metacommunity models can explain community assembly in environments where discrete habitat patches are absent but strong environmental gradients persist. By demonstrating the role of environmental filtering alongside biotic associations, we refine metacommunity predictions for systems with continuous spatial structure and frequent disturbances. Our results support that species sorting is a key process in beach meiofauna community assembly, as both GDM and JSDM analyses revealed that environmental factors are major drivers of community turnover and assembly. However, we also demonstrate that biotic associations contribute significantly to community assembly, advancing the understanding of metacommunity processes by incorporating these factors into models of small‐bodied, understudied taxa. While previous studies indicated meiofauna are influenced by physical factors (Di Domenico et al. 2009; Rodríguez 2007; McLachlan and Defeo 2017; Rodríguez et al. 2001; Rodríguez et al. 2003), our study provides novel insights into how environmental factors and biotic associations interplay locally, revealing a more complex picture of community assembly for hundreds of taxa.

### 4.1 Community Turnover

The GDM results show that the environment is the major driver of community turnover in sandy beaches, with Distance from the Low Tide line being the main factor. The strongest change corresponds to the high tide level, indicating that the transition from marine to terrestrial conditions is the main driver of community turnover. This emphasises the importance of sharp environmental gradients in structuring communities, a phenomenon applicable to other ecosystems with strong habitat transitions. This aligns with previous findings on beach invertebrate communities showing clear differentiation between supralittoral and intertidal areas (Costa, Soares‐Gomes, et al. 2022). Sediment Grain Size was the second most important factor, affecting habitat structure by influencing interstitial space and sediment oxygenation (Fegley et al. 2020; Rodríguez 2007; Rodríguez et al. 2001).

The spatial factor, that is, the distance between sampling sites, was the third most important variable, showing that dispersal limitations play a role in beach meiofauna. This challenges the assumption that microscopic organisms have unlimited dispersal capabilities (Fenchel and Finlay 2004) and highlights the need to consider spatial processes in metacommunity dynamics. Our findings provide empirical evidence that dispersal limitation significantly influences community structure even in widely dispersing organisms, contributing to broader ecological understanding. Previous studies found strong differences in meiofauna dispersal between taxonomic groups (Curini‐Galletti et al. 2012; Vanreusel et al. 2023). While the dispersal of intertidal meiofauna has been studied, there is a lack of research on supralittoral meiofauna, which is important for understanding beach communities holistically. Beach Slope was the fourth important factor, reflecting morphodynamic conditions known to shape communities (McLachlan and Turner 1994). In our area, its effects seem overshadowed by other environmental factors, potentially because most beaches in the study area are of an intermediate or dissipative type. These differences might be more pronounced between reflective and dissipative beaches.

The remaining environmental factors, Mean Surface Salinity and Spring Tide Range, played minor roles. Changes in salinity influenced community turnover between low and medium levels, aligning with findings that many meiofauna are sensitive to salinity changes (Broman et al. 2019; Ingole and Parulekar 1998). Mean Annual Temperature did not influence community turnover, possibly due to homogeneous meteorological conditions across the study area. However, temperature differences might play a role on a smaller scale, for example, due to different currents and beach geomorphology (Kaminski et al. 2021).

### 4.2 Community Assembly Processes

The JSDM results revealed more complex patterns and, in line with Leibold et al. (2022), showed that there is no single dominant driver of community assembly across all sites and species. We found the environmental conditions strongly influence community assembly, especially in sites with distinct conditions, thus supporting our hypothesis. This suggests that meiofauna might occupy narrower abiotic niches in environmentally extreme sites, which could also be in line with the stress gradient hypothesis (Bertness and Callaway 1994). However, against our hypothesis, species sorting due to biotic associations played a significant role, especially in sites that are either very similar or very distinct in their environmental conditions. Biotic interactions may therefore be the key driver of community composition in meiofaunal communities, but it is also possible that we are missing important environmental predictors (Warton et al. 2015). Arguing against the latter is that the spatial component, which could be either a proxy for dispersal processes or a proxy for spatial environmental trends, explains the least of the compositional variation. A more detailed study of within‐beach diversity patterns could provide deeper insights into the drivers of the observed pattern. Such patterns may also be relevant in other ecosystems with sharp environmental gradients or ecotones.

In sites with larger grain sizes, the environmental component was the most important driver, hinting that environmental ‘extremism' for meiofauna communities is mostly defined by grain size, consistent with GDM results. The optimum grain size for intertidal meiofauna appears to be between 200 and 400 μm, corresponding to the findings of (McLachlan and Turner 1994). By quantifying the influence of grain size on community assembly, our study contributes to a more nuanced understanding of habitat suitability for small‐bodied organisms, which can inform ecological models in other sedimentary environments. Similarly, we found that sites with steeper slopes had a higher environmental component for community assembly, indicating that energy levels and habitat stability differ significantly between steep and less steep beaches, leading to increased species sorting due to environmental filtering.

For Spring Tide Range, we found that sites with low ranges had a higher biotic component, suggesting a higher importance of biotic interactions, which supports our hypothesis and metacommunity theory that in less extreme sites, biotic interactions are more important for community assembly, probably because of more competition or facilitation, which would also be in line with the stress gradient hypothesis, while strong hydrodynamic forces act as strong environmental factors for meiofauna (Rodríguez 2007; Gheskiere et al. 2005; McLachlan and Defeo 2017; McLachlan and Turner 1994).

Surprisingly, sites with different levels of salinity did not show strong different community assembly processes, even though GDM analyses indicated a minor influence on community turnover. Other studies found a strong influence of salinity on meiofauna (Baia and Rollnic 2021; Ingole and Parulekar 1998; Lallias et al. 2015), and we suggest that smaller scale changes in salinity across the intertidal zone might be more influential in shaping communities.

While species sorting as a consequence of environmental filtering and biotic associations was dominant, spatial factors played only a minor role, indicating that dispersal limitations do not play a significant role in shaping meiofauna communities. This is in line with the traditional view that small‐bodied organisms have nearly unlimited dispersal capabilities (Fenchel and Finlay 2004). Only for very isolated sites, the spatial component explained more of the compositional variance than the other components (Figure 4), which is to be expected, given that a site or community that is far away from the other sites could also show a different gamma diversity because of different surrounding communities.

The observed spatial structure suggests that while meiofauna can disperse across beaches, the dispersal is insufficient to entirely homogenise communities over the approximately 650 km of coastline included in our study. We observed an interaction between spatial and environmental factors, with spatially unique sites being environmentally distinct. This is plausible, as some sampling sites towards the northern limit of our study area were also among the sites with the coarsest grain size and steepest Beach Slope, while the southernmost sampling sites were among the ones with the shallowest Beach Slope. Since this is inevitable in natural ecosystems, we highlight the need for running controlled experiments to understand the interplay between local and regional processes and the environment, for example, by running controlled field experiments (Emery et al. 2022; Michaud et al. 2019) in different geographic areas to gain a better understanding of the interaction of spatial and environmental factors.

Our JSDM results highlighted that biotic associations play a more important role in community assembly than previously thought, especially in environmentally similar sites. We found that taxa known to occur in the same habitat exhibit the strongest co‐occurrence, such as Copepoda, Platyhelminthes and Polychaeta, which are primarily marine taxa in beach habitats. The decrease in biotic associations with increasing environmental distinctiveness supports the idea that species interactions in beach ecosystems are more pronounced in homogeneous environments (Defeo and McLachlan 2005). This suggests that biotic interactions are key components of community assembly in small‐bodied organisms. Our findings contribute to metacommunity theory by refining predictions for small‐bodied, sediment‐dwelling taxa in highly dynamic environments. Specifically, our study demonstrates that even in spatially continuous landscapes, environmental filtering dominates but does not fully exclude spatial and biotic influences and challenges the assumption that meiofaunal communities are homogenised by high dispersal potential. Biotic interactions are more influential in homogeneous environments, challenging the assumption that small‐bodied organisms are primarily structured by environmental filtering. These insights are particularly relevant for understanding communities of small or microbial taxa, where species sorting is often assumed to dominate without explicit tests of biotic interactions and spatial constraint. However, without sufficient information on the ecological traits of most meiofaunal species, the nature of these interactions remains speculative. Moreover, biotic associations in JSDM can also arise from missing environmental predictors (Dormann et al. 2018).

### 4.3 Implications for Future Research

Our study provides deeper insights into the realised ecological niches of sandy beach meiofauna and the factors shaping their communities. By integrating biotic associations into community models, we contribute to a more comprehensive understanding of community assembly processes, applicable to other ecosystems with small‐bodied, understudied taxa. Previous studies on selected taxa have highlighted the importance of biotic interactions among meiofauna species (Maghsoud et al. 2014; Maria et al. 2012, 2018), but they were more limited in scope. Our approach demonstrates the utility of combining high‐throughput sequencing with advanced statistical modelling to unravel complex ecological processes. Future research should investigate a broad range of meiofaunal taxa to understand their traits, linking them with ecological roles. Metabarcoding and JSDM analyses facilitate niche studies, but more work on selected species is necessary to verify results and understand species interactions.

While this study used the COI marker due to its species‐level resolution and the availability of a curated reference database, future research would benefit from combining multiple genetic markers, such as 18S and COI, to maximise taxonomic coverage and resolution. The complementarity of these markers has been shown to provide broader insights into meiofaunal communities (Castro et al. 2021; Gielings et al. 2021), especially for taxa where a single marker may offer limited resolution (Fais et al. 2020; Fontaneto et al. 2015).

Controlled field and mesocosm experiments are needed to isolate variables and test relationships between environmental variables, spatial factors and species interactions. Although challenging, such experiments can validate findings and enhance understanding of meiofauna responses to different conditions (Elarbaoui et al. 2015; Hockin 1982; Wang et al. 2011; Widbom and Elmgren 1988). Similar experimental approaches could be applied to other ecosystems to test the generality of our findings and to further explore the mechanisms driving community assembly in small‐bodied organisms. This would help to determine whether the patterns observed in meiofauna are consistent across different environments and taxa. For example, experiments with selected pairs of species or communities could test the hypothesis that species interactions are more significant under certain environmental conditions, as has been suggested for sandy beach macrofauna (Costa, Fanini, et al. 2022).

Finally, molecular methods enable the rapid analysis of hundreds of samples. While they need rigorous testing and verification (Blackman et al. 2019; Hakimzadeh et al. 2024; van der Loos and Nijland 2021), they are powerful tools in combination with statistical methods such as JSDM, facilitating analyses of larger and more complex datasets. These approaches can be extended to other ecosystems with rich communities of organisms, enhancing our ability to study biodiversity and community assembly processes across different environments. By leveraging these methods, researchers can overcome traditional challenges associated with studying understudied taxa. For beach ecosystems and meiofauna communities, this enables more comprehensive studies and can help address ecological questions such as to which extent beaches can be considered closed or semi‐closed ecosystems, as suggested by Martínez et al. (2020).

## 5 Conclusion

Our results demonstrate that strong environmental gradients, especially distance from the low tide line and sediment grain size, primarily shape beach meiofauna metacommunities, but no single driver dominates across all sites and taxa. Biotic interactions also contribute significantly to community assembly, particularly under both benign and extreme conditions. Although spatial processes play a lesser role, dispersal limitation emerges in isolated sites, challenging the assumption of entirely unlimited dispersal for small‐bodied organisms. Our findings refine the understanding of metacommunity processes for highly dynamic, continuous habitats by showing how environmental filtering and species interactions jointly structure communities. Future work incorporating trait‐based approaches, additional genetic markers and controlled experiments will further clarify these complex assembly mechanisms and enhance our understanding of small‐bodied, understudied taxa in highly dynamic ecosystems.

## Author Contributions

Jan‐Niklas Macher, Alejandro Martínez, Maximilian Pichler, Simon Creer, Willem Renema and Diego Fontaneto developed the study. Jan‐Niklas Macher and Maximilian Pichler led the data analyses. Maximilian Pichler and Jan‐Niklas Macher led the writing. All authors approved the final version of the manuscript.

## Conflicts of Interest

The authors declare no conflicts of interest.

## Supporting information


Figure S1.

Table S1.

## Data Availability

All raw data is available in the NCBI Sequence Read Archive (SRA); BioProject number PRJNA1081920, as well as in figshare, doi: 10.6084/m9.figshare.26144068.
